# Supplementary material for: Systematic discovery of CRISPR-boosted CAR T cell immunotherapies
Source: Nature. 2025 Sep 24;646(8086):963–72. doi: 10.1038/s41586-025-09507-9 (PMC12545207; doi:10.1038/s41586-025-09507-9)
Supplement: Supplementary file 1 — This file contains Supplementary Note, legends for Supplementary Figs. 1–4 and legends for Supplementary Tables 1–8. [file 41586_2025_9507_MOESM1_ESM.pdf]

nature portfolio

https://doi.org/10.1038/s41586-025-09507-9

Supplementary information

Systematic discovery of CRISPR-boosted CAR T cell immunotherapies

In the format provided by the authors and unedited

Supplementary Note | Scaling the CELLFIE platform for genome-wide multi-readout screens

Genome-wide CRISPR screening in primary CAR T cells requires a large number of gene-edited CAR T cells to be maintained with minimal non-intentional selective pressures or genetic drift. Toward that goal, we conducted careful optimizations of cell culture, lentiviral transduction, mRNA electroporation, cell selection, and screening readouts.

We made several observations and optimizations that helped achieve robust genome-wide screens in CAR T cells. First, CD8 T cells required the presence of CD4 T cells for effective proliferation, while CD4 T cells proliferated better alone (Extended Data Fig. 2c-d). Expectedly, both CD4 and CD8 cells differentiated into effector and memory cells during T cell culture (Extended Data Fig. 3g). Second, we carefully optimized antibiotic selection for cells that were successfully transduced with lentivirus (Extended Data Fig. 2e) and electroporated with synthetic mRNA (Extended Data Fig. 2f). This reduced cell culture costs and enhanced the signal-to-noise ratio in our screens. Third, we scaled our electroporation workflow from small pilot experiments (1.5 million cells per cuvette) to billions of cells with consistent editing efficiency and high cell viability (Extended Data Fig. 2g-h). Fourth, successful lentiviral transduction depended on the stimulation status of T cells, and only a small subset of proliferating cells allowed viral entry. We thus tested widely used TCR stimulation reagents and obtained the best results with ImmunoCult CD3/CD28 activator at day 3 after stimulation (Extended Data Fig. 2i-j). Fifth, we found that the commonly used polybrene reagent was toxic to T cells, whereas spinfection and plate coating with retronectin were well-tolerated but did not further improve T cell transduction (Extended Data Fig. 2j-k).

Using our optimized conditions, transduction rates in primary T cells saturated around 50% for CD4 and 20% for CD8 cells (Extended Data Fig. 2m), which is adequate for genome-wide screening. We quantified lentiviral titers (Extended Data Fig. 2l) and determined lentivirus amounts needed to transduce T cells at a multiplicity of infection of 0.3 (Extended Data Fig. 2m), which was chosen to ensure that only a small percentage of cells receive multiple lentiviral integrations with different gRNAs in the same cell. To accurately quantify the number of gRNA integrations per cell and to confirm our choice of lentiviral amounts, we developed a next generation sequencing assay that is based on single-cell expansion and gRNA amplicon sequencing (Extended Data Fig. 2n). Analyzing 62 T cell clones, we determined an average of 1.5 gRNAs per cell (Extended Data Fig. 2o), which is a fitting value for obtaining a large number of usable cells in genome-wide screens where most gRNAs do not impact the phenotype. Cell numbers for transduction and electroporation were chosen to achieve at least 1000x gRNA coverage in the genome-wide screens (Supplementary Table 2l, 3p). We also confirmed that CAR T cells generated with the optimized conditions expressed the anti-CD19 CAR on their surface (Extended Data Fig. 2p) and achieved highly effective and specific killing of CD19-positive cancer cells (Extended Data Fig. 2q).

Finally, we made several improvements that were critical for genome-wide, multi-readout FACS screening in CAR T cells. First, we developed an efficient sorting strategy to isolate viable CD4 or CD8 CAR T cells following exposure to the target cells (Extended Data Fig. 5e), with pre-selection for those CAR T cells that successfully engaged target cells (evident from trogocytosis-acquired CD19 on their cell surface). Second, we devised a cell fixation protocol that supports prolonged cell sorting without compromising marker staining or cell integrity (Extended Data Fig. 5f-i). This was a critical step, as the total sorting time across all FACS-based screens exceeded 100 hours, and sorting unfixed cells would have been infeasible. Third, complex FACS readouts can result in low numbers of sorted cells, but we demonstrated that the fully optimized workflow can yield high data quality data across multiple genome-wide screens (Supplementary Fig. 3) and in a series of focused validation screens with as few as 1000 sorted cells (Extended Data Fig. 5l-m, Supplementary Table 3).

Supplementary Figure Legends

Supplementary Figure 1 | Timelines of the genome-wide fitness screens.

Experimental timelines for 15 genome-wide fitness screens with TCR or CAR stimulation, indicating exact timing of transduction, electroporation, antibiotic selection, TCR or CAR stimulation, and sample collection.

Supplementary Figure 2 | Timelines of the genome-wide FACS-based screens.

Experimental timelines for 45 genome-wide FACS-based screens with CD19, CD69, FAS, PD1/LAG3/TIM3 readout, indicating the timing and details of transduction, electroporation, antibiotic selection, tumor cell challenge, and sorting.

Supplementary Figure 3 | Quality control of the genome-wide CRISPR screens.

Quality control metrics for all 58 genome-wide CRISPR screening samples analyzed in this study. Shown are the percentage of reads aligning to the gRNA library (NGS library quality), the percentage of gRNAs with zero counts (stochastic dropout), and the Gini index (indicative of selective enrichment).

Supplementary Figure 4 | Design of the *in vivo* CROP-seq method for pooled screening in mice.

a, Cloning of gRNA libraries for *in vivo* CROP-seq. The gRNA library is synthesized as an oligo pool, with the gRNA protospacers flanked by a partial hU6 promoter and the gRNA backbone. The UMI and Illumina Read2 primer are introduced by a separate oligonucleotide. The gRNA oligo pool and the UMI oligo are annealed via their 35-base overlap, extended by a fill-in reaction, and PCR-amplified. CROP-seq vectors are linearized by PCR and assembled with insert by Gibson assembly.

b, Library preparation for *in vivo* CROP-seq, shown here with the CROP-seq-CAR vector. The gRNA expression cassette (consisting of hU6 promoter, gRNA protospacer, UMI, and Illumina TruSeq read 2 primer binding site) is located inside the 3' LTR. During lentiviral integration, the gRNA cassette gets copied to the 5' LTR by viral mechanisms. From the 5' LTR, the gRNA is transcribed by polymerase III from the hU6 promoter for efficient genome editing. The 3' LTR copy is included in the highly expressed CAR-P2A-Puro mRNA. The *in vivo* CROP-seq method detects gRNAs from this high-copy mRNA, rather than from the genomic DNA. It employs construct-specific reverse transcription followed by nested PCR amplification of the gRNA and the UMI. Details for gRNA sequencing on the Illumina NovaSeq 6000 platform are shown at the bottom.

Supplementary Table Legends

Supplementary Table 1 | Plasmid vectors, gRNAs, synthetic oligos, and staining reagents used in this study.

a, Plasmid constructs used for primary CAR T cell and cancer cell line engineering.

b, Plasmid constructs used for synthetic mRNA production.

c, gRNAs used for single-locus targeting, and oligos used for single-locus amplification.

d, Primers used for amplicon sequencing of genomic loci.

e, Primers used for pooled CAR T cell screening.

f, Primers used for *in vivo* CROP-seq cloning, reverse transcription, and outer PCR amplification.

g, Primers used for *in vivo* CROP-seq NGS indexing PCR.

h, Staining reagents for flow cytometry and FACS.

i, Oligonucleotides and gBlocks used for cloning.

j, Primers used for combinatorial CAR T cell screening NGS indexing PCR.

Supplementary Table 2 | CAR T cell fitness screens.

a, Focused gRNA library for the technology validation fitness mini-screens.

b, Sample annotation for the technology validation fitness mini-screens.

c, Raw gRNA counts for the technology validation fitness mini-screens.

d, Genome-wide gRNA library, derived from the Brunello library and cloned into the CROP-seq-CAR vector.

e, Sample annotation for the genome-wide fitness screens.

f, Raw gRNA counts for the genome-wide fitness screens.

g, Quality control metrics for the genome-wide fitness screens.

h, MAGeCK RRA results for the genome-wide fitness screens under TCR stimulation.

i, MAGeCK RRA results for the genome-wide fitness screens under CAR stimulation.

j, Design matrix for MAGeCK MLE analysis of the genome-wide fitness screens.

k, MAGeCK MLE results and beta values for the genome-wide fitness screens with TCR and CAR stimulation.

l, Cell numbers at T cell isolation, transduction, and electroporation.

Supplementary Table 3 | CAR T cell FACS-based screens.

a, Focused gRNA library for the FACS-based mini-screens validating CD69 and FAS readouts.

b, Focused gRNA library for the FACS-based mini-screens validating PD1, LAG3, TIM3 readouts.

c, Sample annotation for the FACS-based mini-screens.

d, Raw gRNA counts for the FACS-based mini-screens validating CD69 and FAS readouts.

e, Raw gRNA counts for the FACS-based mini-screens validating PD1, LAG3, TIM3 readouts.

f, Genome-wide gRNA library, derived from the Brunello library and cloned into the CROP-seq-CAR vector. This library and table are the same as Supplementary Table 2d.

g, Sample annotation for the genome-wide FACS-based screens.

h, Raw gRNA counts for the genome-wide FACS-based screens.

i, MAGeCK RRA results for CD19 negative ($CD19^{-}$) versus unsorted CAR T cells.

j, MAGeCK RRA results for CD19 positive ($CD19^{+}$) versus unsorted CAR T cells.

k, MAGeCK RRA results for CD69 negative ($CD69^{-}$) versus unsorted CAR T cells.

l, MAGeCK RRA results for CD69 positive ($CD69^{+}$) versus unsorted CAR T cells.

m, MAGeCK RRA results for FAS strongly negative (FAS^{---}) versus unsorted CAR T cells.

n, MAGeCK RRA results for PD1, LAG3, TIM3 triple-negative versus unsorted CAR T cells.

o, MAGeCK RRA results for PD1, LAG3, TIM3 triple-positive versus unsorted CAR T cells.

p, Cell numbers at T cell isolation, transduction, and electroporation.

q, Quality control metrics for genome-wide FACS screens.

Supplementary Table 4 | Comparison with published T cell and CAR T cell screening datasets.

a, Overview of published genome-wide screens in human T cells and CAR T cells.

b, Comparison of screening quality based on the separation of essential and non-essential genes.

c, Log_2 fold changes for essential and non-essential gene knockouts compared across studies.

d, MLE beta values for essential and non-essential gene knockouts compared across studies.

e, MLE beta values for *PDCD1* and non-essential gene knockouts in PD1 sorting screens compared across studies.

f, Overview of screening hits for each study.

g, Integration of screening hits across all high-quality datasets.

Supplementary Table 5 | *In vivo* screens in a xenograft mouse model of human leukemia.

a, Development of *in vivo* CROP-seq and comparison to standard pooled screening.

b, Focused gRNA library for the *in vivo* CROP-seq screens.

c, Sample annotation for the *in vivo* CROP-seq screens.

d, Raw gRNA counts for the *in vivo* CROP-seq screens.

e, Raw gRNA counts for the *in vivo* CROP-seq screens with 16 UMI-based internal replicates.

f, Results of the *in vivo* CROP-seq screens.

g, Number of T cell clones detected in each organ.

h, Off-target predictions for the gRNA targeting FAS and RHOG.

Supplementary Table 6 | RNA-seq analysis of RHOG knockout versus standard CAR T cells.

a, Sample annotation for the RNA-seq analysis.

b, Normalized and filtered RNA-seq read counts.

c, Differential gene expression for RHOG knockout versus standard (safe harbor locus-edited) CAR T cells.

d, Gene set enrichment analysis (GSEA) for Gene Ontology (GO) Biological Process terms.

Supplementary Table 7 | Combinatorial screens for CAR T cell fitness.

a, gRNA library for the combinatorial screens.

b, Sample annotation for the combinatorial screens.

c, Raw gRNA counts for the combinatorial screens.

d, Quality control metrics for the combinatorial screens.

e, MLE design matrix for the analysis of the combinatorial screens with MAGeCK MLE.

f, MAGeCK MLE results for the combinatorial screens.

Supplementary Table 8 | Base editing screens for RHOG and various controls.

a, gRNA library for the tiling base editing screen with the predicted mutations for each gRNA.

b, Sample annotation for the tiling base editing screen.

c, Raw gRNA counts for the tiling base editing screen.

d, MAGeCK RRA results for Day 12 versus Day 0 of the tiling base editing screen.

e, gRNA library for the validation base editing screen with the predicted mutations for each gRNA.

f, Sample annotation for the validation base editing screen.

g, Raw gRNA counts for the validation base editing screen.
