# Supplementary material for: Systematic discovery of CRISPR-boosted CAR T cell immunotherapies
Source: Nature. 2025 Sep 24;646(8086):963–72. doi: 10.1038/s41586-025-09507-9 (PMC12545207; doi:10.1038/s41586-025-09507-9)
Supplement: Supplementary file 2 — Reporting Summary [file 41586_2025_9507_MOESM2_ESM.pdf]

nature portfolio

Corresponding author(s): Paul Datlinger, Christoph Bock

Last updated by author(s): Jul 29, 2025

Reporting Summary

Nature Portfolio wishes to improve the reproducibility of the work that we publish. This form provides structure for consistency and transparency in reporting. For further information on Nature Portfolio policies, see our Editorial Policies and the Editorial Policy Checklist.

Statistics

For all statistical analyses, confirm that the following items are present in the figure legend, table legend, main text, or Methods section.

n/a	Confirmed	
☐	☒	The exact sample size (n) for each experimental group/condition, given as a discrete number and unit of measurement
☐	☒	A statement on whether measurements were taken from distinct samples or whether the same sample was measured repeatedly
☐	☒	The statistical test(s) used AND whether they are one- or two-sided<br>*Only common tests should be described solely by name; describe more complex techniques in the Methods section.*
☒	☐	A description of all covariates tested
☐	☒	A description of any assumptions or corrections, such as tests of normality and adjustment for multiple comparisons
☐	☒	A full description of the statistical parameters including central tendency (e.g. means) or other basic estimates (e.g. regression coefficient) AND variation (e.g. standard deviation) or associated estimates of uncertainty (e.g. confidence intervals)
☐	☒	For null hypothesis testing, the test statistic (e.g. F, t, r) with confidence intervals, effect sizes, degrees of freedom and P value noted<br>*Give P values as exact values whenever suitable.*
☒	☐	For Bayesian analysis, information on the choice of priors and Markov chain Monte Carlo settings
☒	☐	For hierarchical and complex designs, identification of the appropriate level for tests and full reporting of outcomes
☒	☐	Estimates of effect sizes (e.g. Cohen's d, Pearson's r), indicating how they were calculated

Our web collection on statistics for biologists contains articles on many of the points above.

Software and code

Policy information about availability of computer code

Data collection

Sequencing: Illumina NovaSeq 6000 platform
Flow cytometry and cell sorting: BD LSRFortessa, Sony SH800S, BD FACSAria Fusion
In vivo bioluminescence imaging: IVIS Spectrum In Vivo Imaging System (PerkinElmer #124262)
In vitro bioluminescence imaging: Perkin Elmer Victor x3 2030 Multilabel Reader

Data analysis

The data analysis is described in detail in the Methods, including includes the following sections:
- Data pre-processing for the CRISPR screens
- Analysis of the genome-wide fitness screens
- Analysis of the genome-wide FACS-based screens
- Analysis of the in vivo CROP-seq screens
- RNA-seq data processing
- Differential expression and gene set enrichment analysis
- Analysis of the combinatorial screens
- Comparison of screening quality with published datasets
- Comparison of screening results with published datasets
- Base editing analysis

Software used: FlowJo v10.810.0, Living Image v4.7.3, Aura In Vivo Imaging Software v4.5.0; software packages used in the custom analysis code are indicated in the relevant sections of the Methods.

For manuscripts utilizing custom algorithms or software that are central to the research but not yet described in published literature, software must be made available to editors and reviewers. We strongly encourage code deposition in a community repository (e.g. GitHub). See the Nature Portfolio guidelines for submitting code & software for further information.

Data

Policy information about availability of data

All manuscripts must include a data availability statement. This statement should provide the following information, where applicable:

- Accession codes, unique identifiers, or web links for publicly available datasets
- A description of any restrictions on data availability
- For clinical datasets or third party data, please ensure that the statement adheres to our policy

The RNA-seq data are available from GEO (accession number: GSE266618). The CRISPR screening data are provided in Supplementary Table 2 (fitness screens), Supplementary Table 3 (FACS screens), Supplementary Table 5 (in vivo screens), Supplementary Table 7 (combinatorial screens), and Supplementary Table 8 (base editing screens).

Research involving human participants, their data, or biological material

Policy information about studies with human participants or human data. See also policy information about sex, gender (identity/presentation), and sexual orientation and race, ethnicity and racism.

Reporting on sex and gender	N/A
Reporting on race, ethnicity, or other socially relevant groupings	N/A
Population characteristics	N/A
Recruitment	N/A
Ethics oversight	N/A

Note that full information on the approval of the study protocol must also be provided in the manuscript.

Field-specific reporting

Please select the one below that is the best fit for your research. If you are not sure, read the appropriate sections before making your selection.

☒ Life sciences ☐ Behavioural & social sciences ☐ Ecological, evolutionary & environmental sciences

For a reference copy of the document with all sections, see nature.com/documents/nr-reporting-summary-flat.pdf

Life sciences study design

All studies must disclose on these points even when the disclosure is negative.

Sample size	Sample size was based on established standards in the field, including the number of independent biological experiments and the number of blood samples from healthy human donors for CAR T cell production (n≥3 unless otherwise indicated). For the in vivo experiments, the determination of group sizes and the power calculation have been done according to the animal license (BMWFW-2020-0.605.586).
Data exclusions	No data were excluded from the manuscript.
Replication	Fitness and FACS-based genome-wide screens were performed in at least 2 independent experiments and with least 3 independent T cell donors, as detailed in Supplementary Table 2 and 3. In vivo experiments for the validation of RHOG knockout CAR T cells were performed in 3 independent experiments using 6 independent T cells donors, as detailed in Supplementary Table 6. All experiments were replicated in at least 2 independent T cell donors, and the number is indicated. No replicates failed or had to be excluded from the analysis.
Randomization	Processing of samples from in vivo screening or validation experiments did not follow any particular order. Due to the uniform and fast engraftment of NALM6 cells, randomization for the NALM6 Xenograft model was not performed. To reduce biases introduced by spill-over during bioluminescence imaging, we aimed to image treatment groups together. Mouse experiments with the Huh7 solid tumor xenograft model were randomized prior to CAR T injection according to tumor size to ensure all groups had equivalent tumor load prior to treatment.<br><br>Age and sex-matched mice were used for all experiments and were maintained in a standardized location with standardized handling procedures and husbandry conditions. Experiments were performed at a consistent time of day to control for circadian effects.
Blinding	Investigators were not blinded to the treatment status during experiments as they also conducted the outcome assessment. Stringent, objective criteria were used for data collection and analysis to mitigate bias.

Reporting for specific materials, systems and methods

We require information from authors about some types of materials, experimental systems and methods used in many studies. Here, indicate whether each material, system or method listed is relevant to your study. If you are not sure if a list item applies to your research, read the appropriate section before selecting a response.

Materials & experimental systems

n/a	Involved in the study
☐	☒ Antibodies
☐	☒ Eukaryotic cell lines
☒	☐ Palaeontology and archaeology
☐	☒ Animals and other organisms
☒	☐ Clinical data
☒	☐ Dual use research of concern
☒	☐ Plants

Methods

n/a	Involved in the study
☒	☐ ChIP-seq
☐	☒ Flow cytometry
☒	☐ MRI-based neuroimaging

Antibodies

Antibodies used

Antibodies and other staining reagents used in this study are listed in Supplementary Table 1 and in the Methods.

The following antibodies were used:
CD4 PerCP/Cyanine5.5 RPA-T4 Biolegend #300530
CD8 PerCP/Cyanine5.5 SK1 Biolegend #344710
CD19 BV605 HIB19 Biolegend #302243
CD19 PE-Cy7 HIB19 Biolegend #302216
CD69 PE-Cy7 FN50 Biolegend #310912
FAS (CD95) PE-Cy7 DX2 Biolegend #305622
PD1 PE-Cy7 EH12.2H7 Biolegend #329918
LAG3 PE-Cy7 11C3C65 Biolegend #369310
TIM3 PE-Cy7 F38-2E2 Biolegend #345014
TIGIT (VSTM3) PerCP-Cy 5.5 A15153G Biolegend #372717
CD279 (PD-1) BV605 EH12.2H7 Biolegend #329923
CD223 (LAG-3) AF647 11C3C65 Biolegend #369303
CD62L PE DREG-56 Biolegend #304806
CD45RO PerCP-Cy5.5 UCHL1 Biolegend #304221
CD107a PE-Cy7 H4A3 Biolegend #328618

Each antibody is reported by the manufacturer as being validated for specificity in flow cytometry on human samples, as described in the product data sheets and on the manufacturer's website. These antibodies are widely used and have been referenced in peer-reviewed literature for the indicated application and species. No further in-house validation was performed.

Validation

All antibodies were validated by the supplier.

Eukaryotic cell lines

Policy information about cell lines and Sex and Gender in Research

Cell line source(s)

NALM6 and K562 cell lines were obtained from the American Type Culture Collection (ATCC), and were engineered to express transgenes as described in the Methods section "Cancer cell lines". The Huh7 cell line was a gift from the Giulio Superti-Furga Lab (CeMM). The NALM-6-GD2 cell line was a gift from the Chrystal Mackall Lab (Stanford).

Authentication

Cell line authentication was performed for the NALM6, NALM6-GD2, and K562 cell lines using Cell Line Identification service (Eurofins), which provided DNA (STR) profile results consistent with expected cells lines. The Huh7 cell line was authenticated by the Giulio Superti-Furga Lab (CeMM).

Mycoplasma contamination

All cell lines repeatedly tested negative for mycoplasma contamination.

Commonly misidentified lines
(See ICLAC register)

The cell lines used are not listed as commonly misidentified at the ICLAC register.

Animals and other research organisms

Policy information about studies involving animals; ARRIVE guidelines recommended for reporting animal research, and Sex and Gender in Research

Laboratory animals

NOD/SCID/IL-2Rγ-null (NSG) mice were bred and maintained under specific-pathogen-free conditions at the Medical University of

Laboratory animals	Vienna. In vivo experiments were performed at the Core Facility Laboratory for Animal Breeding and Husbandry. Age-matched male or female mice (8 to 12 weeks) were used, and all experiments were performed in individually ventilated cages at ambient temperature and humidity according to the application of the Medical University of Vienna for the authorization of breeders, suppliers and users under no. BMWFW-66.009/0403-WF/V/3b/2014 at a 12 hour light/dark cycle.
Wild animals	No wild animals were used in this study.
Reporting on sex	Female mice were used for in vivo screens with the NALM6 xenograft model and the Huh7 solid tumor xenograft model. Male mice were used for individual validation of CAR T cells with single knockouts in the NALM6 xenograft model.
Field-collected samples	No field-collected samples were used in this study.
Ethics oversight	All experiments were performed according to the animal experiment license BMWFW-2020-0.605.586, granted by the Austrian Federal Ministry of Education, Science and Research (BMBWF) as licensing committee. All experiments were approved by the institutional ethical committee at the Department for Biomedical Research of the Medical University of Vienna and followed institutional guidelines.

Note that full information on the approval of the study protocol must also be provided in the manuscript.

Plants

Seed stocks	N/A
Novel plant genotypes	N/A
Authentication	N/A

Flow Cytometry

Plots

Confirm that:

☒ The axis labels state the marker and fluorochrome used (e.g. CD4-FITC).

☒ The axis scales are clearly visible. Include numbers along axes only for bottom left plot of group (a 'group' is an analysis of identical markers).

☒ All plots are contour plots with outliers or pseudocolor plots.

☒ A numerical value for number of cells or percentage (with statistics) is provided.

Methodology

Sample preparation	Cells were stained as follows: Pellet the cells, wash with 1x PBS, stain with 1/1000 Zombie Viability Dye (Biolegend) at room temperature for 10 min, wash and pellet the cells, stain with a mix of the relevant antibodies in Cell Staining Buffer (Biolegend #420201) at 4 ̊C for 30 min, wash and pellet cells, resuspend in the desired volume, and filter through a cell strainer. For storage prior to cell sorting, cells were fixed in Fluorofix Buffer (Biolegend #422101) at room temperature for 30 min in the dark, followed by washing cells twice and storing them in Cell Staining Buffer at a concentration of 50 million/ml. For large-scale screens, staining and fixation steps were done in 50 ml tubes placed on a rotator to avoid cell pelleting and clumping. The staining reagents used in this study are listed in Supplementary Table 1.
Instrument	For flow cytometry profiling, the BD LSRFortessa cell analyzer was used. For cell sorting, the Sony SH800S cell sorter was used for optimization experiments and the BD FACSAria Fusion for genome-wide screening.
Software	Data analysis was performed with FlowJo (Version 10.10.0) software.
Cell population abundance	The number of cells collected and processed in FACS-based genome-wide screens is reported in Supplementary Table 3 for each individual sample (in the range of 15,085-64,000,000 cells, depending on the sample and in particular the selected percentage of marker-positive or marker-negative cells).
Gating strategy	The gating strategy for FACS-based genome-wide screens is depicted in Extended Data Fig. 5e and described in Supplementary Table 3 for each individual sample. Gating was performed on all cells (using FSC-A/SSC-A axes), alive cells (live/dead stain-negative), CD4+ or CD8+ cells to select T cells, then single-cell events (using FSC-A/FSC-W axes), and then assessed the expression of individual markers used for profiling and/or cell sorting.

☒ Tick this box to confirm that a figure exemplifying the gating strategy is provided in the Supplementary Information.
