# Supplementary figures and images for: Systematic discovery of CRISPR-boosted CAR T cell immunotherapies
Source: Nature. 2025 Sep 24;646(8086):963–72. doi: 10.1038/s41586-025-09507-9 (PMC12545207; doi:10.1038/s41586-025-09507-9)

Timelines of the genome-wide fitness screens

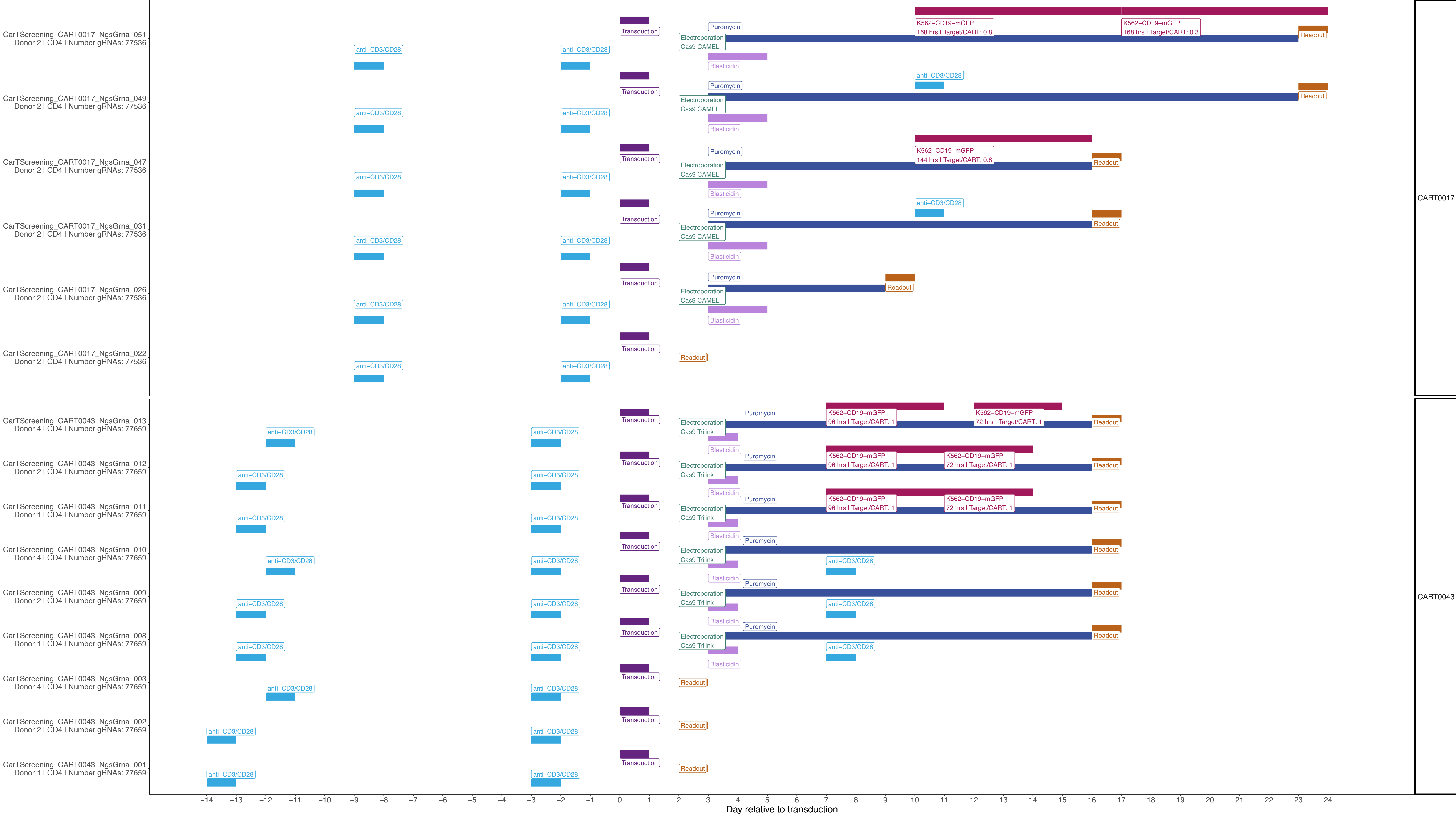

Supplement: Supplementary file 3 — Timelines of the genome-wide fitness screens. See Supplementary Information for full legend. [file 41586_2025_9507_MOESM3_ESM.pdf]

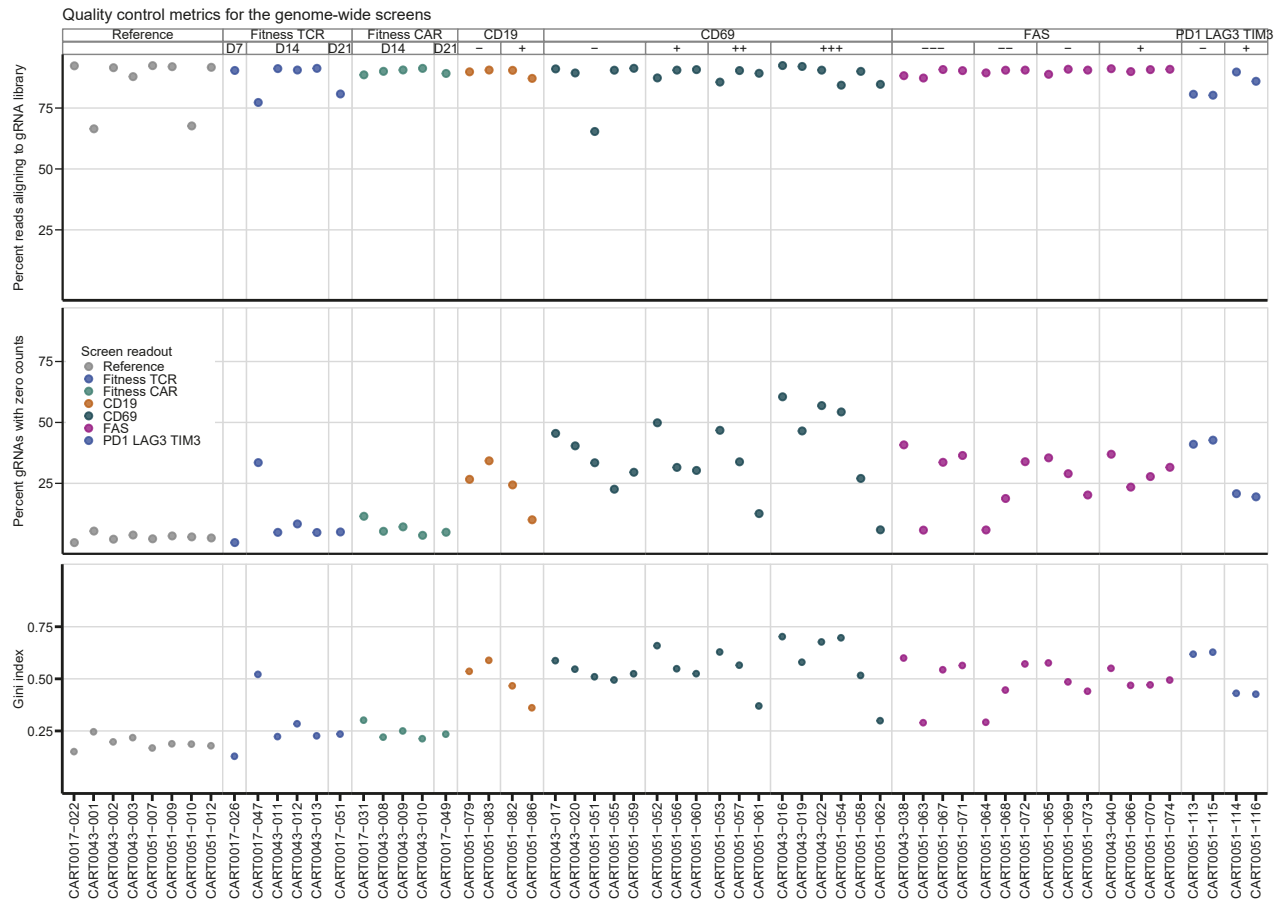

Supplement: Supplementary file 5 — Quality control of genome-wide CRISPR screens. See Supplementary Information for full legend. [file 41586_2025_9507_MOESM5_ESM.pdf]
