# Supplementary material for: Systematic discovery of CRISPR-boosted CAR T cell immunotherapies
Source: Nature. 2025 Sep 24;646(8086):963–72. doi: 10.1038/s41586-025-09507-9 (PMC12545207; doi:10.1038/s41586-025-09507-9)
Supplement: Supplementary file 6 — Design of the in vivo CROP-seq method for pooled screening in mice. See Supplementary Information for full legend. [file 41586_2025_9507_MOESM6_ESM.pdf]

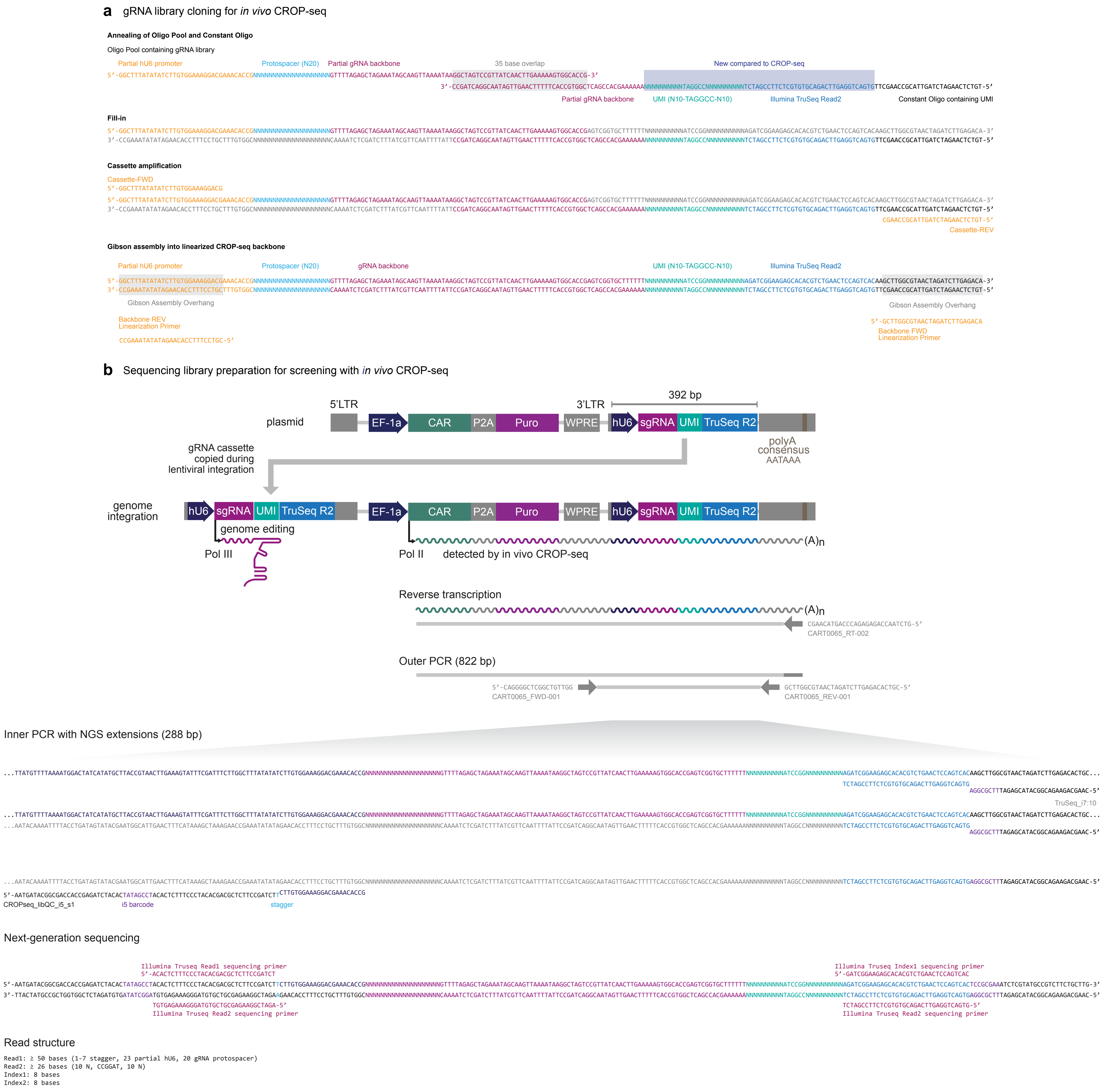
a gRNA library cloning for in vivo CROP-seq
Annealing of Oligo Pool and Constant Oligo
Oligo Pool containing gRNA library
Partial hU6 promoter
Protospacer (N20)
Partial gRNA backbone
35 base overlap
New compared to CROP-seq
Partial gRNA backbone
UMI (N10-TAGGCC-N10)
Illumina TruSeq Read2
Constant Oligo containing UMI
Fill-in
Cassette amplification
Cassette-FWD
5'-GGCTTTATATATCTTGTGGAAAGGACG
CGAACCGCATTGATCTAGAACTCTGT-5'
Cassette-REV
Gibson assembly into linearized CROP-seq backbone
Partial hU6 promoter
Protospacer (N20)
gRNA backbone
UMI (N10-TAGGCC-N10)
Illumina TruSeq Read2
Gibson Assembly Overhang
Gibson Assembly Overhang
Backbone REV
Linearization Primer
CCGAAATATATAGAACACCTTTCCTGC-5'
5'-GCTTGGCGTAACTAGATCTTGAGACA
Backbone FWD
Linearization Primer
b Sequencing library preparation for screening with in vivo CROP-seq
plasmid
5'LTR
EF-1a
CAR
P2A
Puro
WPRE
3'LTR
392 bp
hU6
sgRNA
UMI
TruSeq R2
polyA consensus AATAAA
gRNA cassette copied during lentiviral integration
genome integration
genome editing
Pol III
Pol II
detected by in vivo CROP-seq
(A)n
Reverse transcription
CGAACATGACCCAGAGAGACCAATCTG-5'
CART0065_RT-002
Outer PCR (822 bp)
5'-CAGGGGCTCGGCTGTTGG
CART0065_FWD-001
GCTTGGCGTAACTAGATCTTGAGACACTGC-5'
CART0065_REV-001
Inner PCR with NGS extensions (288 bp)
TruSeq_i7:10
CROPseq_libQC_i5_s1
i5 barcode
stagger
Next-generation sequencing
Illumina Truseq Read1 sequencing primer
Illumina Truseq Index1 sequencing primer
Illumina Truseq Read2 sequencing primer
Illumina Truseq Read2 sequencing primer
Read structure
Read1: ≥ 50 bases (1-7 stagger, 23 partial hU6, 20 gRNA protospacer)
Read2: ≥ 26 bases (10 N, CCGGAT, 10 N)
Index1: 8 bases
Index2: 8 bases
