# Supplementary material for: Systematic discovery of CRISPR-boosted CAR T cell immunotherapies
Source: Nature. 2025 Sep 24;646(8086):963–72. doi: 10.1038/s41586-025-09507-9 (PMC12545207; doi:10.1038/s41586-025-09507-9)

Timelines of the genome-wide FACS-based screens

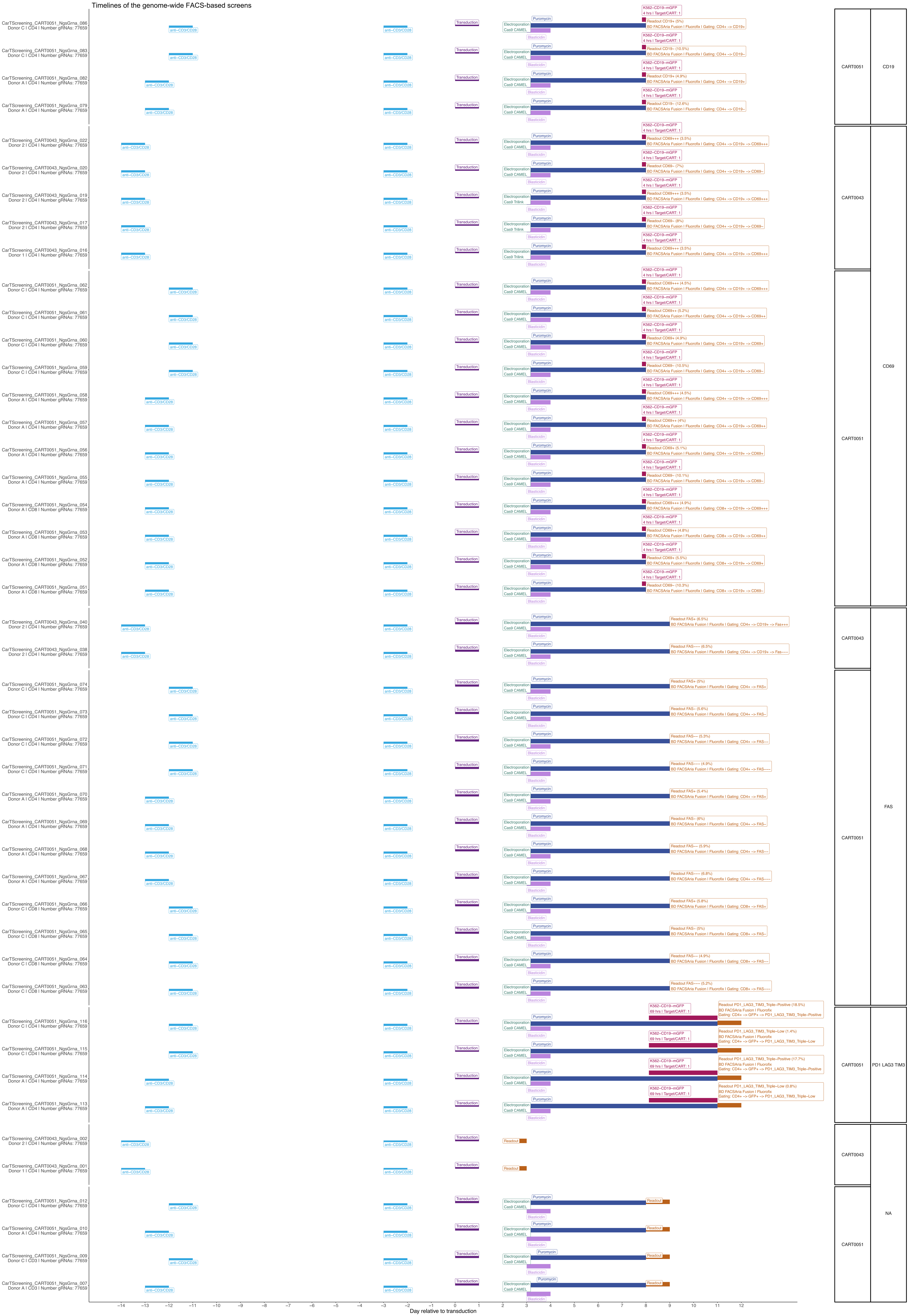

Supplement: Supplementary file 4 — Timelines of the genome-wide FACS-based screens. See Supplementary Information for full legend. [file 41586_2025_9507_MOESM4_ESM.pdf]
